# Crystal structure of the 1:1 co-crystal 4-(di­methylamino)­pyridin-1-ium 8-hy­droxy­quinoline-5-sulfonate–*N*,*N*-di­methyl­pyridin-4-amine

**DOI:** 10.1107/S205698902400642X

**Published:** 2024-07-09

**Authors:** Mami Isobe, Yukiyasu Kashiwagi, Koji Kubono

**Affiliations:** a Department of Chemistry and Bioengineering, Graduate School of Engineering, Osaka Metropolitan University, 3-3-138 Sugimoto, Sumiyoshi-ku, Osaka 558-8585, Japan; b Osaka Research Institute of Industrial Science and Technology, 1-6-50, Morinomiya, Joto-ku, Osaka 536-8553, Japan; c Osaka Kyoiku University, 4-698-1 Asahigaoka Kashiwara Osaka 582-8582 Japan; University of Hyogo, Japan

**Keywords:** crystal structure, co-crystal, quinolin-8-ol sulfonate, DMAP, N—H⋯N inter­actions

## Abstract

The asymmetric unit of the title compound is composed of two independent ion pairs of 4-(di­methyl­amino)­pyridin-1-ium 8-hy­droxy­quinoline-5-sulfonate (HDMAP^+^·HqSA^−^, C_7_H_11_N_2_^+^·C_9_H_6_NO_4_S^−^) and neutral *N*,*N*-di­methyl­pyridin-4-amine mol­ecules (DMAP, C_7_H_10_N_2_), co-crystallized as a 1:1:1 HDMAP^+^:HqSA^−^:DMAP adduct in the monoclinic system, space group *Pc*. The compound has a layered structure, including cation layers of HDMAP^+^ with DMAP and anion layers of HqSA^−^ in the crystal. In the cation layer, there are inter­molecular N—H⋯N hydrogen bonds between the protonated HDMAP^+^ mol­ecule and the neutral DMAP mol­ecule. In the anion layer, each HqSA^−^ is surrounded by other six HqSA^−^, where the planar network structure is formed by inter­molecular O—H⋯O and C—H⋯O hydrogen bonds. The cation and anion layers are linked by inter­molecular C—H⋯O hydrogen bonds and C—H⋯*π* inter­actions.

## Abstract 2

The asymmetric unit of the title compound consists of two independent ion pairs of 4-(di­methyl­amino)­pyridin-1-ium quinolin-8-ol-5-sulfonate (HDMAP^+^·HqSA^−^) and neutral *N*,*N*-di­methyl­pyridin-4-amine (DMAP), forming a 1:1:1 cation:anion:neutral mol­ecule co-crystal. The compound has a layered structure, including cation layers of HDMAP^+^ with DMAP and anion layers of HqSA^−^ in the crystal. The cation and anion layers are linked by inter­molecular C—H⋯O hydrogen bonds and C—H⋯*π* inter­actions.

## 1. Chemical context

Ionic co-crystals have much attention in pharmaceuticals for the development of improved drugs based on crystal engin­eering (Bolla *et al.*, 2022 ▸) and in organic functional materials for achieving rare and multifunctional properties through tunable structures, morphologies, and sizes in co-crystal assemblies (Sun *et al.*, 2019 ▸). In structural chemistry, ionic co-crystals containing pyridine-pyridinium derivatives bridged by an N—H⋯N hydrogen bond have already been proposed (Doring & Jones, 2016 ▸; Fabry *et al.*, 2017 ▸; Zhang *et al.*, 2018 ▸; Vladiskovic *et al.*, 2023 ▸). In addition, the supra­molecular synthon preference of pyridinium salts to 8-hy­droxy­quinoline-5-sulfonate (HqSA^−^) and various sulfonates has been investigated (Ganie *et al.*, 2021 ▸). On the other hand, quinolin-8-ol and its sulfonated derivative, quinoline-8-ol sulfonic acid (H_2_qSA), are well-known chelating ligands and analytical reagents (Wiberley *et al.*, 1949 ▸; Kashiwagi *et al.*, 2020 ▸; Kubono *et al.*, 2023 ▸). H_2_qSA shows higher solubility to water than quionolin-8-ol, especially under basic conditions. We report here the crystal structure of the title compound as an ionic co-crystal composed of the salt of 4-(di­methyl­amino)­pyridin-1-ium (HDMAP^+^) and quinolin-8-ol-5-sulfonate (HqSA^−^) with neutral *N*,*N*-di­methyl­pyridin-4-amine (DMAP).

## 2. Structural commentary

**Figure 1:**
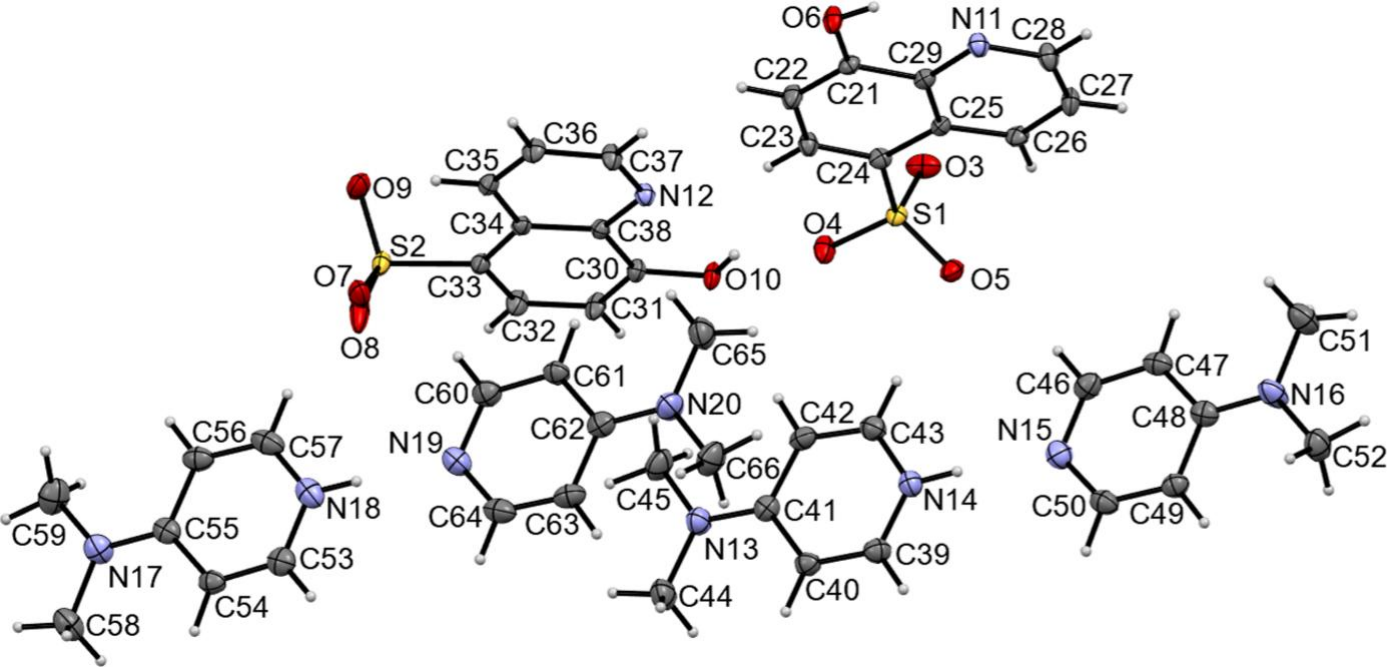
The mol­ecular structure of the title compound with atom labeling. Displacement ellipsoids are drawn at the 50% probability level. H atoms are represented by spheres of arbitrary radius.

The title compound is composed of two independent HDMAP^+^·HqSA^−^ ion pairs and neutral DMAP mol­ecules, co-crystallized in the monoclinic system, space group *Pc* as shown in Fig. 1 ▸. The phenolic H atoms (H6, H10) in the HqSA^−^ moieties are not dissociated.
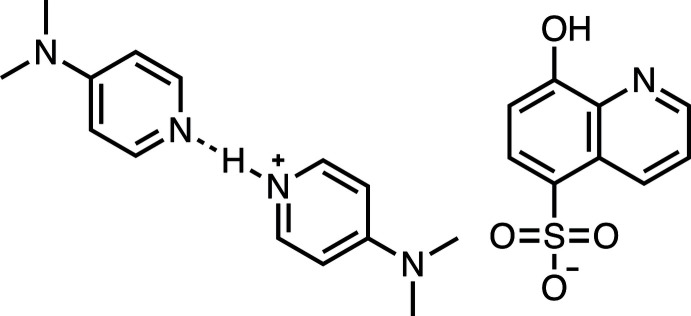


**Figure 2:**
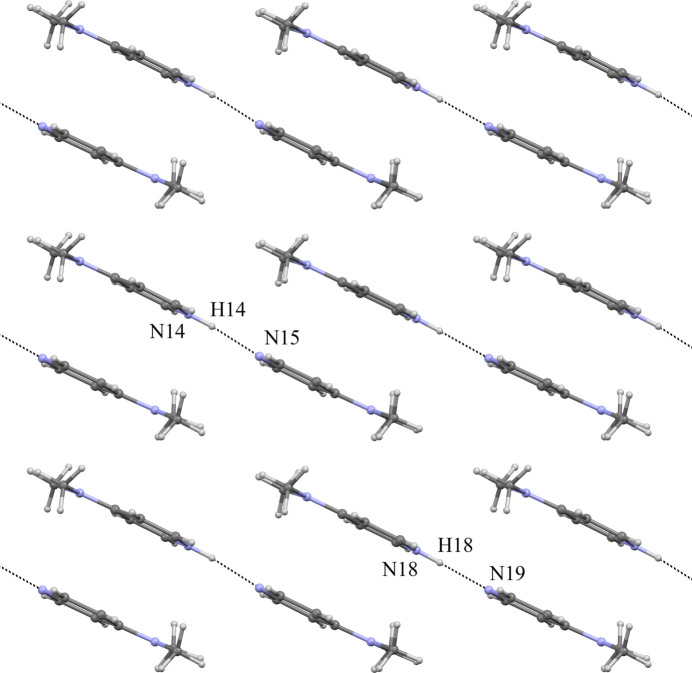
The layer structure of the [HDMAP·DMAP]^+^ cationic unit in the *ab* plane. The inter­molecular N—H⋯N hydrogen bonds are shown as dashed lines.

There are intra­molecular O—H⋯N hydrogen bonds involving the hy­droxy groups and quinoline N atoms (O6—H6⋯N11 and O10—H10⋯N12; Table 1 ▸) generating *S*(5) ring motifs (Fig. 2 ▸). The proton of the sulfonate group in H_2_qSA is dissociated and bound to the pyridyl N atom of one DMAP mol­ecule, but there is also another non-protonated DMAP mol­ecule in the crystal. As a result, the co-crystal is formulated as a 1:1:1 HDMAP^+^:HqSA^−^:DMAP adduct. The cations of HDMAP^+^ are formed through inter­molecular N14—H14⋯N15 and N18—H18⋯N19 hydrogen bonds in a linear geometry (Fig. 2 ▸, see below). Each H atom attached to the N atom of the pyridine ring in HDMAP^+^ could be located in a Fourier density map, and the N14—H14 and N18—H18 bond lengths are similar, 0.90 (3) Å. The N atoms of the di­methyl­amino groups (N13, N16, N17 and N20) show no pyramidalization, with deviations from the plane of the bonded three C atoms of 0.029 (7), 0.031 (3), 0.037 (8) and 0.020 (4) Å, respectively. The quinoline ring systems in HqSA^−^ are essentially planar, the dihedral angles between the mean planes of the pyridine and benzene rings N12/C34–C38 and C30—C34/C38, and N11/C25–C29 and C21–C25/C29 being 0.46 (14) and 0.78 (13)°, respectively.

## 3. Supra­molecular features

**Figure 3:**
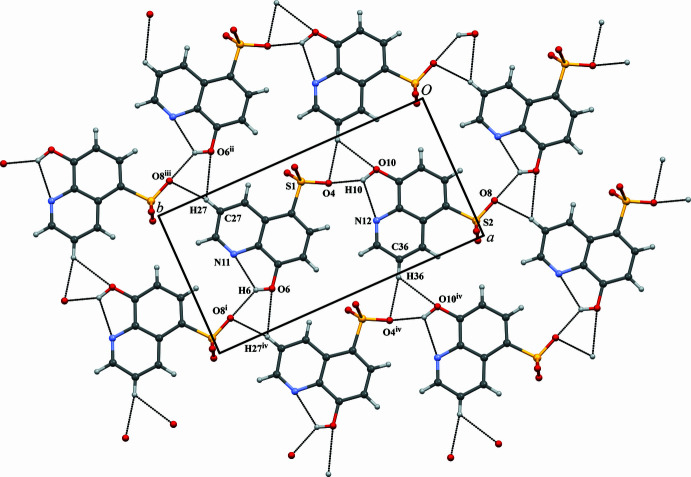
The *S*(5) ring motifs formed by intra­molecular O—H⋯N hydrogen bonds involving the hy­droxy groups and quinoline N atoms of the HqSA^−^ anionic units. The intra­molecular O—H⋯N hydrogen bonds are shown as dashed lines. The sheet structure of the HqSA^−^ anionic units is formed by the planar inter­molecular hydrogen-bond networks in the *ab* plane. The inter­molecular O—H⋯O, C—H⋯O, O—H⋯N hydrogen bonds are also shown as dashed lines. [Symmetry codes: (i) *x*, *y* − 1, *z*; (ii) *x* − 1, *y, z*; (iii) *x* − 1, *y* − 1, *z*; (iv) *x* + 1, *y*, *z*.].

**Figure 4:**
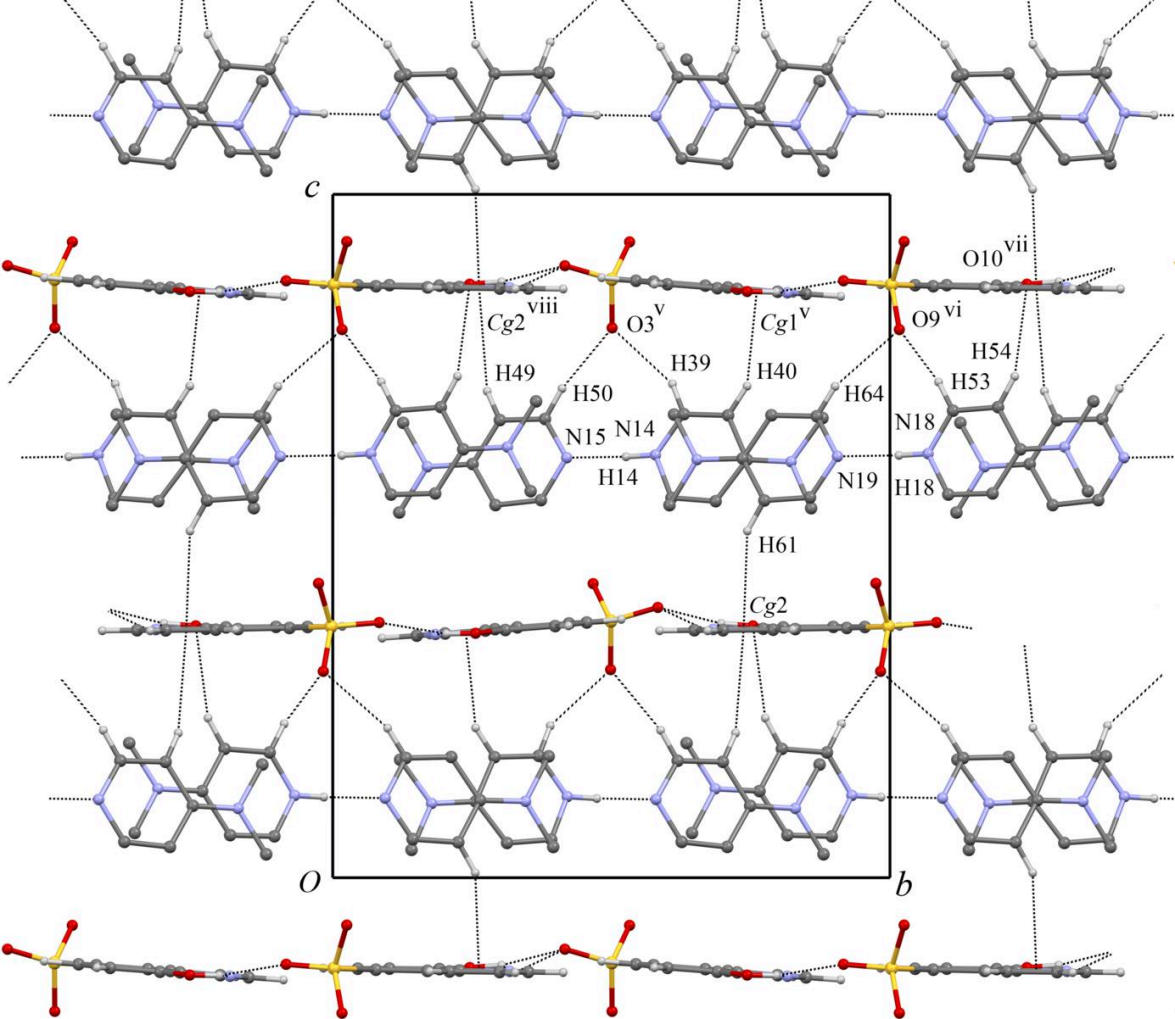
The network structure between [HDMAP·DMAP]^+^ cationic layers and HqSA^−^ anion layers. The inter­molecular C—H⋯O hydrogen bonds and C—H⋯*π* inter­actions are shown as dashed lines. The 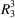
(8) motifs of independent ion pairs formed by an inter­molecular N—H⋯N hydrogen bond and two inter­molecular C—H⋯O hydrogen bonds are also shown as dashed lines. [Symmetry codes: (v) *x*, −*y* + 1, *z* + 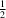; (vi) *x*, −*y* + 2, *z* + 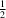; (vii) *x* + 1, −*y* + 2, *z* + 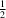; (viii) *x* − 1, −*y* + 1, *z* + 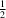.].

In the title co-crystal, both the cation layers of [HDMAP·DMAP]^+^ and the anion layers of HqSA^−^ run parallel to the *ab* plane. The hydrogen-bond geometry is summarized in Table 1 ▸. The pyridine rings in the cation layer are stacked along the *ab* plane as shown in Fig. 2 ▸. In the cation layer, two independent cation units of [HDMAP·DMAP]^+^ are formed by inter­molecular N—H⋯N hydrogen bonds (N14—H14⋯N15 and N18—H18⋯N19). The N14—H14⋯N15 and N18—H18⋯N19 angles are 174 (3) and 177 (7)°, respectively. The dihedral angles between the two pyridine rings in the [HDMAP·DMAP]^+^ units are 0.21 (15)° (N14/C39–C43 and N15/C46–C50 rings) and 1.60 (15)° (N18/C53–C57 and N19/C60–C64). The quinoline ring system in the anion layer faces the *ab* plane as shown in Fig. 3 ▸. In the anion layer, each HqSA^−^ mol­ecule is surrounded by six HqSA^−^ mol­ecules through inter­molecular hydrogen bonds, essentially forming an sheet. Each HqSA^−^ mol­ecule binds with two HqSA^−^ mol­ecules having the same mol­ecular orientation through inter­molecular C—H⋯O hydrogen bonds [C27—H27⋯O6^ii^ and C36—H36⋯O10^iv^; symmetry codes: (ii) *x* − 1, *y*, *z*; (iv) *x* + 1, *y*, *z*] and also binds with four HqSA^−^ mol­ecules having the different mol­ecular orientation through inter­molecular O—H⋯O and C—H⋯O hydrogen bonds [O6—H6⋯O8^i^, O10—H10⋯O4, C27—H27⋯O8^iii^ and C36—H36⋯O4^iv^; symmetry codes: (i) *x*, *y* − 1, *z*; (iii) *x* − 1, *y* − 1, *z*]. The C27—H27⋯O6^ii^, C36—H36⋯O10^iv^, O6—H6⋯O8^i^, O10—H10⋯O4, C27—H27⋯O8^iii^ and C36—H36⋯O4^iv^ angles are 125, 125, 137 (4), 144 (4), 164 and 160°, respectively. The inter­planar spacing between adjacent anionic layers (the distance between the closest centroids of the mean planes through N12/C22/C23/C37 within the anionic layers, being across the cationic layer from each other) is 9.562 Å. The inter­actions between the cationic and anionic layers are attributed to the extended 3D hydrogen-bonding linkages, three C—H⋯*π* inter­actions [C40—H40⋯*Cg*1^i^, C49—H49⋯*Cg*2^viii^, C61—H61⋯*Cg*2; *Cg*1 and *Cg*2 are the centroids of the N11/C25–C29 and N12/C34–C38 rings, respectively; symmetry code: (viii) *x* − 1, 1 − *y*, *z* + 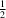] and five C—H⋯O inter­actions [C39—H39⋯O3^v^, C50—H50⋯O3^v^, C53—H53⋯O9^vi^, C54—H54⋯O10^vii^, C64—H64⋯O9^vi^; symmetry code: (v) *x*, 1 − *y*, *z* + 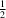; (vi) *x*, 2 − *y*, *z* + 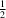; (vii) *x* + 1, 2 − *y*, *z* + 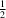] as shown in Fig. 4 ▸ and Table 1 ▸. In addition, each independent ion pair forms 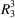
(8) motif by one inter­molecular N—H⋯N hydrogen bond and two inter­molecular C—H⋯O hydrogen bonds (N14—H14⋯N15, C39—H39⋯O3^v^ and C50—H50⋯O3^v^; N18—H18⋯N19, C53—H53⋯O9^vi^ and C64—H64⋯O9^vi^).

## 4. Database survey

A search of the Cambridge Structural Database (CSD, Version 2024.1.0, update of March 2024; Groom *et al.*, 2016 ▸) for compounds containing the 4-amino­pyridine skeleton with hydrogen atom bound at the 2, 3, 5, 6-positions of the pyridine ring gave 5687 hits. Among those, a search for the containing DMAP mol­ecule gave 1794 hits and for those of protonated DMAP gave 360 hits. A search for compounds containing a pyridine-protonated pyridine skeleton gave 15 hits. In these compounds, the dihedral angles between two pyridine rings are close to 0° in seven structures, which are essentially co-planar due to unique hydrogen-bonding networks stemming from the substituents on the pyridine rings (BAYBIN; Kobayashi *et al.*, 2003 ▸; BECHOG; Glidewell *et al.*, 1982 ▸; KIFBIO; Vladiskovic *et al.*, 2023 ▸; WAZNET; Lackova *et al.*, 2014 ▸; WEVHOX; Zhang *et al.*, 2018 ▸; XACFOW; Mautner & Goher, 1998 ▸; XOHWAT; Santra *et al.*, 2008 ▸). In single crystals of salts of the mellitate anion, which is obtained by deprotonation of mellitic acid (benzene hexa­carb­oxy­lic acid), with substituted pyridinium derivatives, the triangular hydrogen-bonded unit between the anions induces a two-dimensional sheet self-organizing structure (BAYBIN, Kobayashi *et al.*, 2003 ▸). On the other hand, ferrocene derivatives substituted with pyridine form cationic dimers *via* a hydrogen bond between two pyridine rings (WOFGII; Braga *et al.*, 2008 ▸). A search for containing both of protonated DMAP and the other neutral DMAP gave 14 hits. There are five hits having the proton between two *N*-(4-pyrid­yl)di­methyl­amine skeletons (2, 3, 5, 6-carbon atoms are bound to hydrogen atoms). In these compounds, the dihedral angles between two pyridine rings are close to 0° in three structures, which are essentially co-planar structures [1.3 (1)° in FETDEO, Aakeroy *et al.*, 2005 ▸; 3.47 (7)° in GOFRUQ, Wagler *et al.*, 2014 ▸; 3.8 (4)° in ZAPNIN, Biradha *et al.*, 1995 ▸]. A fragment search for the 8-hy­droxy­quinoline-5-sulfonic acid skeleton gave 84 hits, which include two hydrate co-crystals composed of the 8-hy­droxy­quinoline-5-sulfonicin anion and 4-phenyl­pyridine (EMEDUY; Ganie *et al.*, 2021 ▸), 4,4′-bipyrydine (INEMAP; Baskar Raj *et al.*, 2003 ▸) cations and three hydrate co-crystals composed of the 8-hy­droxy-7-iodo­quinoline-5-sulfonic anion and various pyridine derivative cations (EFAQUZ, Smith *et al.*, 2012 ▸; EYIYOA, Smith *et al.*, 2004 ▸; ISUTAR, Hemamalini *et al.*, 2004 ▸). According to the crystal structures of BAYBIN, EFAQUZ, EYIYOA and ISUTAR, these compounds form layered structures by constructing 2D layers of the cationic and anionic moieties with these layers arranged sterically.

## 5. Synthesis and crystallization

To a solution of DMAP (611 mg, 5.0 mmol) in H_2_O (5 mL) at 353 K, an ethanol (1 mL) solution of H_2_qSA (450 mg, 2.0 mmol) was added and then stirred for 30 min. Orange single crystals of the title compound suitable for X-ray diffraction were grown by slow evaporation of the aqueous ethanol solution mentioned above for a week at ambient temperature.

## 6. Refinement

Crystal data, data collection and structure refinement details are summarized in Table 2 ▸. The title compound was refined as an inversion twin in *Pc* whose twin component mass ratio refined to 0.522 (18):0.478 (18). The hy­droxy H atoms, H6 and H10, were located in a difference-Fourier map and freely refined. The N-bound H atoms, H14 and H18, were located in difference-Fourier maps but were refined with a distance restraint of N—H = 0.86 ± 0.02 Å. All H atoms bound to carbon were positioned geometrically and refined using a riding model, with C—H = 0.95 or 0.98 Å and *U*_iso_(H) = 1.2 or 1.5*U*_eq_(C).

## Supplementary Material

Crystal structure: contains datablock(s) I. DOI: 10.1107/S205698902400642X/ox2006sup1.cif

Structure factors: contains datablock(s) I. DOI: 10.1107/S205698902400642X/ox2006Isup3.hkl

Supporting information file. DOI: 10.1107/S205698902400642X/ox2006Isup3.cml

CCDC reference: 2366836

Additional supporting information:  crystallographic information; 3D view; checkCIF report

## Figures and Tables

**Table 1:** Hydrogen-bond geometry (Å, °) *Cg*1, *Cg*2 are the centroids of the N11/C25–C29 and N12/C34–C38 rings, respectively

*D*—H⋯*A*	*D*—H	H⋯*A*	*D*⋯*A*	*D*—H⋯*A*
O6—H6⋯O8^i^	0.84 (5)	2.00 (5)	2.679 (3)	137 (4)
O6—H6⋯N11	0.84 (5)	2.24 (5)	2.728 (3)	117 (4)
O10—H10⋯O4	0.89 (5)	1.90 (5)	2.674 (3)	144 (4)
O10—H10⋯N12	0.89 (5)	2.31 (5)	2.730 (3)	109 (4)
N14—H14⋯N15	0.90 (3)	1.91 (3)	2.814 (4)	174 (3)
N18—H18⋯N19	0.90 (3)	1.92 (4)	2.816 (4)	177 (7)
C27—H27⋯O6^ii^	0.95	2.58	3.219 (4)	125
C27—H27⋯O8^iii^	0.95	2.27	3.194 (4)	164
C36—H36⋯O4^iv^	0.95	2.33	3.244 (4)	160
C36—H36⋯O10^iv^	0.95	2.58	3.216 (4)	125
C39—H39⋯O3^v^	0.95	2.32	3.200 (4)	154
C43—H43⋯O5	0.95	2.22	3.160 (4)	169
C46—H46⋯O5	0.95	2.46	3.373 (4)	161
C50—H50⋯O3^v^	0.95	2.43	3.292 (4)	151
C53—H53⋯O9^vi^	0.95	2.22	3.146 (4)	164
C54—H54⋯O10^vii^	0.95	2.55	3.450 (4)	158
C57—H57⋯O7	0.95	2.22	3.143 (4)	165
C60—H60⋯O7	0.95	2.40	3.325 (4)	163
C64—H64⋯O9^vi^	0.95	2.35	3.267 (4)	162
C40—H40⋯*Cg*1^v^	0.95	2.63	3.498 (3)	153
C49—H49⋯*Cg*2^viii^	0.95	2.87	3.754 (3)	156
C61—H61⋯*Cg*2	0.95	2.70	3.571 (3)	152

Symmetry codes: (i) 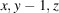; (ii) 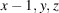; (iii) 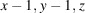; (iv) 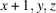; (v) 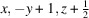; (vi) 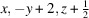; (vii) 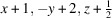; (viii) 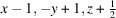.

**Table 2:** Experimental details

Crystal data	
Chemical formula	C_7_H_11_N_2_^+^·C_9_H_6_NO_4_S^−^·C_7_H_10_N_2_
*M*_r_	469.55
Crystal system, space group	Monoclinic, *Pc*
Temperature (K)	100
*a*, *b*, *c* (Å)	8.00032 (10), 15.14469 (18), 18.9141 (2)
β (°)	100.6050 (12)
*V* (Å^3^)	2252.53 (5)
*Z*	4
Radiation type	Cu *K*α
μ (mm^−1^)	1.62
Crystal size (mm)	0.4 × 0.30 × 0.11

Data collection	
Diffractometer	XtaLAB Synergy, Dualflex, HyPix
Absorption correction	Multi-scan (*CrysAlis PRO*; Rigaku OD, 2023 ▸)
*T*_min_, *T*_max_	0.731, 1.000
No. of measured, independent and observed [*I* > 2σ(*I*)] reflections	16358, 6813, 6608
*R*_int_	0.030
(sin θ/λ)_max_ (Å^−1^)	0.632

Refinement	
*R*[*F*^2^ > 2σ(*F*^2^)], *wR*(*F*^2^), *S*	0.033, 0.085, 1.04
No. of reflections	6813
No. of parameters	620
No. of restraints	4
H-atom treatment	H atoms treated by a mixture of independent and constrained refinement
Δρ_max_, Δρ_min_ (e Å^−3^)	0.30, −0.39
Absolute structure	Refined as an inversion twin
Absolute structure parameter	0.478 (18)

Computer programs: *CrysAlis PRO* (Rigaku OD, 2023 ▸), *SHELXT2018/2* (Sheldrick, 2015*a* ▸), *SHELXL2018/3* (Sheldrick, 2015*b* ▸) and *OLEX2* 1.5 (Dolomanov *et al.*, 2009 ▸).

## supplementary crystallographic information

### 4-(Dimethylamino)pyridin-1-ium 8-hydroxyquinoline-5-sulfonate; N,N-dimethylpyridin-4-amine Crystal data

**Table:**

C_7_H_11_N_2_^+^·C_9_H_6_NO_4_S^−^·C_7_H_10_N_2_	*F*(000) = 992
*M**_r_* = 469.55	*D*_x_ = 1.385 Mg m^−^^3^
Monoclinic, *P**c*	Cu *K*α radiation, λ = 1.54184 Å
*a* = 8.00032 (10) Å	Cell parameters from 11719 reflections
*b* = 15.14469 (18) Å	θ = 3.8–76.8°
*c* = 18.9141 (2) Å	µ = 1.62 mm^−^^1^
β = 100.6050 (12)°	*T* = 100 K
*V* = 2252.53 (5) Å^3^	Block, colourless
*Z* = 4	0.4 × 0.30 × 0.11 mm

### 4-(Dimethylamino)pyridin-1-ium 8-hydroxyquinoline-5-sulfonate; N,N-dimethylpyridin-4-amine Data collection

**Table:**

XtaLAB Synergy, Dualflex, HyPix diffractometer	6813 independent reflections
Radiation source: micro-focus sealed X-ray tube, PhotonJet (Cu) X-ray Source	6608 reflections with *I* > 2σ(*I*)
Mirror monochromator	*R*_int_ = 0.030
Detector resolution: 10.0000 pixels mm^-1^	θ_max_ = 77.2°, θ_min_ = 3.8°
ω scans	*h* = −10→9
Absorption correction: multi-scan (CrysAlisPro; Rigaku OD, 2023)	*k* = −17→19
*T*_min_ = 0.731, *T*_max_ = 1.000	*l* = −22→23
16358 measured reflections	

### 4-(Dimethylamino)pyridin-1-ium 8-hydroxyquinoline-5-sulfonate; N,N-dimethylpyridin-4-amine Refinement

**Table:**

Refinement on *F*^2^	Hydrogen site location: mixed
Least-squares matrix: full	H atoms treated by a mixture of independent and constrained refinement
*R*[*F*^2^ > 2σ(*F*^2^)] = 0.033	*w* = 1/[σ^2^(*F*_o_^2^) + (0.0463*P*)^2^ + 0.5548*P*] where *P* = (*F*_o_^2^ + 2*F*_c_^2^)/3
*wR*(*F*^2^) = 0.085	(Δ/σ)_max_ < 0.001
*S* = 1.04	Δρ_max_ = 0.30 e Å^−^^3^
6813 reflections	Δρ_min_ = −0.39 e Å^−^^3^
620 parameters	Absolute structure: Refined as an inversion twin
4 restraints	Absolute structure parameter: 0.478 (18)
Primary atom site location: dual	

### 4-(Dimethylamino)pyridin-1-ium 8-hydroxyquinoline-5-sulfonate; N,N-dimethylpyridin-4-amine Special details

**Table:**

Geometry. All esds (except the esd in the dihedral angle between two l.s. planes) are estimated using the full covariance matrix. The cell esds are taken into account individually in the estimation of esds in distances, angles and torsion angles; correlations between esds in cell parameters are only used when they are defined by crystal symmetry. An approximate (isotropic) treatment of cell esds is used for estimating esds involving l.s. planes.
Refinement. Refined as a 2-component inversion twin.1. Twinned data refinement Scales: 0.522 (18) 0.478 (18) 2. Fixed Uiso At 1.2 times of: All C(H) groups At 1.5 times of: All C(H,H,H) groups 3. Restrained distances H18-N18 0.86 with sigma of 0.02 H14-N14 0.86 with sigma of 0.02 4.a Aromatic/amide H refined with riding coordinates: C35(H35), C23(H23), C27(H27), C26(H26), C49(H49), C31(H31), C28(H28), C61(H61), C54(H54), C36(H36), C60(H60), C50(H50), C22(H22), C40(H40), C53(H53), C37(H37), C42(H42), C47(H47), C64(H64), C63(H63), C46(H46), C43(H43), C39(H39), C32(H32), C56(H56), C57(H57) 4.b Idealised Me refined as rotating group: C52(H52A,H52B,H52C), C44(H44A,H44B,H44C), C58(H58A,H58B,H58C), C66(H66A,H66B, H66C), C51(H51A,H51B,H51C), C45(H45A,H45B,H45C), C65(H65A,H65B,H65C), C59(H59A, H59B,H59C)

### 4-(Dimethylamino)pyridin-1-ium 8-hydroxyquinoline-5-sulfonate; N,N-dimethylpyridin-4-amine Fractional atomic coordinates and isotropic or equivalent isotropic displacement parameters (Å^2^)

**Table:**

	*x*	*y*	*z*	*U*_iso_*/*U*_eq_	
S1	0.17772 (8)	0.49921 (4)	0.37557 (4)	0.01431 (15)	
S2	0.88938 (8)	0.99725 (4)	0.36763 (4)	0.01686 (16)	
O5	0.0868 (3)	0.46728 (13)	0.43037 (11)	0.0198 (4)	
O10	0.3080 (3)	0.75413 (13)	0.36853 (12)	0.0189 (4)	
O4	0.2618 (3)	0.58342 (12)	0.39552 (12)	0.0222 (4)	
O7	1.0147 (3)	0.97468 (16)	0.43079 (12)	0.0274 (5)	
O6	0.7498 (3)	0.25632 (14)	0.35776 (12)	0.0191 (4)	
N11	0.4365 (3)	0.18427 (15)	0.35601 (13)	0.0168 (5)	
O3	0.0728 (3)	0.49865 (13)	0.30454 (12)	0.0251 (5)	
N14	0.0994 (3)	0.57921 (17)	0.61872 (14)	0.0228 (5)	
O9	0.9547 (3)	0.98318 (15)	0.30206 (12)	0.0253 (5)	
N18	1.0806 (4)	1.06837 (18)	0.62048 (15)	0.0283 (6)	
N12	0.6186 (3)	0.68257 (14)	0.36322 (13)	0.0153 (5)	
N17	1.3114 (3)	1.31360 (18)	0.63022 (14)	0.0268 (6)	
N13	0.3275 (3)	0.82252 (17)	0.60874 (14)	0.0248 (6)	
C25	0.3141 (4)	0.33136 (18)	0.36637 (15)	0.0132 (5)	
N16	−0.3146 (4)	0.16966 (18)	0.60144 (14)	0.0300 (6)	
N15	−0.0827 (3)	0.41978 (17)	0.61461 (14)	0.0258 (6)	
C30	0.4422 (3)	0.80852 (18)	0.36639 (15)	0.0152 (6)	
N20	0.6851 (4)	0.65381 (18)	0.61189 (15)	0.0297 (6)	
C29	0.4524 (4)	0.27401 (18)	0.36172 (15)	0.0141 (5)	
N19	0.9083 (4)	0.90581 (18)	0.61654 (15)	0.0271 (6)	
C35	0.9054 (4)	0.79031 (19)	0.36300 (15)	0.0157 (5)	
H35	1.003375	0.825760	0.363038	0.019*	
O8	0.8170 (3)	1.08342 (14)	0.37345 (18)	0.0427 (7)	
C48	−0.2420 (4)	0.25088 (19)	0.60531 (16)	0.0222 (6)	
C23	0.5063 (4)	0.45581 (18)	0.37350 (16)	0.0165 (6)	
H23	0.525964	0.517580	0.378068	0.020*	
C24	0.3458 (4)	0.42408 (18)	0.37218 (15)	0.0156 (6)	
C27	0.1385 (4)	0.20164 (19)	0.35746 (16)	0.0195 (6)	
H27	0.031056	0.173969	0.355266	0.023*	
C26	0.1533 (4)	0.29119 (19)	0.36372 (15)	0.0162 (6)	
H26	0.056312	0.326366	0.366290	0.019*	
C34	0.7451 (3)	0.82997 (18)	0.36388 (15)	0.0137 (5)	
C49	−0.2102 (4)	0.29987 (19)	0.66961 (16)	0.0224 (6)	
H49	−0.243039	0.277078	0.711817	0.027*	
C31	0.4187 (4)	0.89824 (19)	0.36710 (17)	0.0192 (6)	
H31	0.309000	0.921934	0.367659	0.023*	
C38	0.6054 (4)	0.77244 (17)	0.36430 (15)	0.0130 (5)	
C28	0.2840 (4)	0.15080 (18)	0.35428 (16)	0.0197 (6)	
H28	0.271473	0.088498	0.350666	0.024*	
C33	0.7176 (4)	0.92240 (18)	0.36554 (15)	0.0152 (5)	
C61	0.8018 (4)	0.7746 (2)	0.55202 (16)	0.0215 (6)	
H61	0.781003	0.744043	0.507393	0.026*	
C54	1.1908 (4)	1.1940 (2)	0.68862 (16)	0.0220 (6)	
H54	1.212304	1.223795	0.733567	0.026*	
C36	0.9166 (4)	0.70039 (19)	0.36211 (16)	0.0184 (6)	
H36	1.022730	0.672608	0.361413	0.022*	
C62	0.7562 (4)	0.7356 (2)	0.61344 (17)	0.0237 (6)	
C60	0.8763 (4)	0.8566 (2)	0.55657 (17)	0.0257 (7)	
H60	0.907308	0.880142	0.514258	0.031*	
C50	−0.1315 (4)	0.3809 (2)	0.67126 (17)	0.0247 (6)	
H50	−0.110204	0.411581	0.715822	0.030*	
C22	0.6432 (4)	0.39925 (19)	0.36825 (17)	0.0193 (6)	
H22	0.753237	0.423009	0.368649	0.023*	
C40	0.2142 (4)	0.7096 (2)	0.67653 (16)	0.0209 (6)	
H40	0.240263	0.743534	0.719363	0.025*	
C53	1.1159 (4)	1.1131 (2)	0.68335 (18)	0.0257 (7)	
H53	1.087382	1.087116	0.725275	0.031*	
C37	0.7696 (4)	0.64896 (18)	0.36221 (17)	0.0183 (6)	
H37	0.780438	0.586507	0.361522	0.022*	
C42	0.2165 (4)	0.6872 (2)	0.55097 (16)	0.0229 (6)	
H42	0.243782	0.705189	0.506333	0.027*	
C41	0.2560 (4)	0.74334 (19)	0.61167 (16)	0.0200 (6)	
C21	0.6173 (4)	0.30979 (18)	0.36257 (15)	0.0145 (5)	
C47	−0.1930 (4)	0.2920 (2)	0.54526 (16)	0.0237 (6)	
H47	−0.212812	0.263269	0.499838	0.028*	
C64	0.8639 (4)	0.8686 (2)	0.67528 (18)	0.0296 (7)	
H64	0.885457	0.901387	0.718843	0.036*	
C55	1.2371 (4)	1.23419 (19)	0.62685 (16)	0.0218 (6)	
C52	−0.3516 (5)	0.1263 (2)	0.66566 (19)	0.0318 (7)	
H52A	−0.246320	0.120190	0.701170	0.048*	
H52B	−0.399916	0.067773	0.652889	0.048*	
H52C	−0.433271	0.161935	0.686096	0.048*	
C63	0.7900 (4)	0.7873 (2)	0.67674 (17)	0.0282 (7)	
H63	0.761539	0.765609	0.720146	0.034*	
C44	0.3650 (5)	0.8790 (2)	0.67269 (19)	0.0302 (7)	
H44A	0.258673	0.894730	0.688314	0.045*	
H44B	0.422323	0.932840	0.661072	0.045*	
H44C	0.439097	0.847174	0.711421	0.045*	
C46	−0.1165 (4)	0.3737 (2)	0.55256 (17)	0.0250 (6)	
H46	−0.085302	0.399369	0.511048	0.030*	
C43	0.1398 (4)	0.6079 (2)	0.55592 (17)	0.0238 (6)	
H43	0.113795	0.571602	0.514381	0.029*	
C39	0.1381 (4)	0.6302 (2)	0.67832 (17)	0.0230 (6)	
H39	0.111083	0.609654	0.722336	0.028*	
C32	0.5578 (4)	0.95491 (19)	0.36698 (17)	0.0202 (6)	
H32	0.541065	1.016967	0.367926	0.024*	
C56	1.2007 (4)	1.1840 (2)	0.56237 (17)	0.0252 (6)	
H56	1.230849	1.206924	0.519661	0.030*	
C58	1.3420 (5)	1.3650 (2)	0.69703 (19)	0.0322 (7)	
H58A	1.233475	1.377047	0.712141	0.048*	
H58B	1.397149	1.420969	0.688945	0.048*	
H58C	1.415965	1.331447	0.734677	0.048*	
C57	1.1241 (4)	1.1046 (2)	0.56067 (18)	0.0292 (7)	
H57	1.100001	1.073059	0.516531	0.035*	
C66	0.6549 (5)	0.6125 (2)	0.6777 (2)	0.0355 (8)	
H66A	0.761279	0.610761	0.712932	0.053*	
H66B	0.613111	0.552185	0.667276	0.053*	
H66C	0.569870	0.646643	0.697307	0.053*	
C51	−0.3476 (5)	0.1193 (2)	0.53443 (18)	0.0345 (8)	
H51A	−0.429289	0.151412	0.498551	0.052*	
H51B	−0.394504	0.061445	0.543216	0.052*	
H51C	−0.241090	0.111422	0.516567	0.052*	
C45	0.3605 (5)	0.8585 (2)	0.54079 (18)	0.0316 (7)	
H45A	0.434078	0.817948	0.520221	0.047*	
H45B	0.416725	0.916003	0.549571	0.047*	
H45C	0.252586	0.865738	0.507093	0.047*	
C65	0.6452 (5)	0.6043 (2)	0.54515 (19)	0.0320 (7)	
H65A	0.569908	0.639435	0.509079	0.048*	
H65B	0.588361	0.549017	0.553627	0.048*	
H65C	0.750475	0.591031	0.527717	0.048*	
C59	1.3515 (5)	1.3552 (2)	0.5656 (2)	0.0355 (8)	
H59A	1.429459	1.317305	0.545016	0.053*	
H59B	1.405242	1.412637	0.578214	0.053*	
H59C	1.246532	1.363609	0.530309	0.053*	
H14	0.047 (5)	0.5262 (17)	0.616 (2)	0.029 (9)*	
H10	0.336 (6)	0.697 (3)	0.372 (2)	0.037 (11)*	
H18	1.027 (6)	1.016 (2)	0.618 (3)	0.057 (15)*	
H6	0.714 (6)	0.204 (3)	0.357 (2)	0.039 (12)*	

### 4-(Dimethylamino)pyridin-1-ium 8-hydroxyquinoline-5-sulfonate; N,N-dimethylpyridin-4-amine Atomic displacement parameters (Å^2^)

**Table:**

	*U*^11^	*U*^22^	*U*^33^	*U*^12^	*U*^13^	*U*^23^
S1	0.0135 (3)	0.0121 (3)	0.0179 (3)	0.0021 (2)	0.0043 (2)	0.0012 (2)
S2	0.0140 (3)	0.0113 (3)	0.0270 (4)	−0.0012 (2)	0.0083 (3)	−0.0004 (2)
O5	0.0221 (10)	0.0170 (9)	0.0228 (11)	0.0007 (8)	0.0104 (8)	−0.0003 (8)
O10	0.0124 (10)	0.0129 (10)	0.0327 (12)	−0.0020 (8)	0.0079 (8)	0.0022 (8)
O4	0.0193 (10)	0.0115 (9)	0.0376 (12)	0.0007 (8)	0.0100 (9)	−0.0005 (8)
O7	0.0270 (12)	0.0331 (11)	0.0216 (11)	−0.0145 (10)	0.0027 (9)	−0.0034 (9)
O6	0.0117 (10)	0.0142 (10)	0.0321 (12)	0.0006 (8)	0.0054 (8)	0.0000 (8)
N11	0.0166 (11)	0.0136 (11)	0.0202 (12)	0.0000 (9)	0.0030 (9)	0.0005 (9)
O3	0.0270 (12)	0.0286 (12)	0.0185 (11)	0.0128 (9)	0.0012 (9)	0.0007 (8)
N14	0.0237 (13)	0.0207 (12)	0.0239 (14)	−0.0030 (10)	0.0041 (10)	−0.0013 (10)
O9	0.0251 (11)	0.0316 (11)	0.0205 (11)	−0.0083 (9)	0.0076 (9)	0.0026 (9)
N18	0.0251 (14)	0.0240 (14)	0.0335 (16)	0.0010 (11)	−0.0006 (11)	−0.0050 (11)
N12	0.0142 (11)	0.0127 (11)	0.0188 (12)	−0.0001 (9)	0.0029 (9)	−0.0001 (9)
N17	0.0303 (14)	0.0248 (13)	0.0229 (13)	−0.0001 (11)	−0.0010 (11)	0.0038 (10)
N13	0.0297 (14)	0.0218 (13)	0.0231 (14)	−0.0044 (10)	0.0052 (11)	0.0012 (10)
C25	0.0125 (12)	0.0147 (13)	0.0128 (12)	−0.0001 (10)	0.0035 (10)	0.0009 (10)
N16	0.0447 (17)	0.0249 (13)	0.0179 (13)	−0.0064 (12)	−0.0008 (12)	−0.0001 (10)
N15	0.0263 (14)	0.0231 (13)	0.0275 (14)	−0.0001 (11)	0.0036 (11)	−0.0002 (10)
C30	0.0120 (13)	0.0159 (13)	0.0178 (14)	−0.0007 (11)	0.0029 (10)	0.0012 (11)
N20	0.0382 (16)	0.0294 (14)	0.0225 (14)	−0.0068 (12)	0.0081 (12)	0.0030 (10)
C29	0.0135 (13)	0.0158 (13)	0.0132 (13)	0.0016 (10)	0.0029 (10)	0.0012 (10)
N19	0.0289 (15)	0.0246 (13)	0.0264 (14)	−0.0010 (11)	0.0014 (11)	−0.0038 (10)
C35	0.0134 (13)	0.0174 (13)	0.0165 (14)	−0.0010 (11)	0.0035 (10)	0.0008 (10)
O8	0.0219 (12)	0.0115 (10)	0.100 (2)	−0.0016 (9)	0.0251 (13)	−0.0010 (12)
C48	0.0210 (15)	0.0243 (15)	0.0199 (15)	0.0037 (12)	−0.0001 (12)	0.0005 (12)
C23	0.0158 (12)	0.0119 (13)	0.0220 (14)	−0.0004 (11)	0.0037 (11)	0.0004 (10)
C24	0.0167 (14)	0.0138 (13)	0.0167 (14)	0.0028 (11)	0.0039 (11)	0.0014 (10)
C27	0.0133 (13)	0.0160 (13)	0.0295 (16)	−0.0032 (11)	0.0045 (11)	0.0022 (11)
C26	0.0157 (14)	0.0149 (13)	0.0190 (14)	0.0032 (10)	0.0057 (11)	−0.0009 (10)
C34	0.0128 (13)	0.0163 (13)	0.0119 (12)	−0.0005 (11)	0.0023 (10)	−0.0003 (10)
C49	0.0241 (15)	0.0249 (15)	0.0182 (14)	0.0009 (12)	0.0039 (11)	0.0013 (11)
C31	0.0110 (14)	0.0170 (13)	0.0308 (17)	0.0032 (11)	0.0070 (11)	0.0025 (11)
C38	0.0131 (13)	0.0130 (12)	0.0129 (12)	−0.0002 (10)	0.0026 (10)	0.0004 (10)
C28	0.0178 (14)	0.0130 (13)	0.0274 (16)	−0.0008 (10)	0.0017 (12)	0.0014 (11)
C33	0.0144 (13)	0.0148 (13)	0.0172 (14)	−0.0010 (11)	0.0048 (10)	0.0006 (10)
C61	0.0237 (15)	0.0239 (15)	0.0160 (14)	−0.0009 (12)	0.0015 (11)	−0.0010 (11)
C54	0.0199 (14)	0.0267 (15)	0.0185 (14)	0.0022 (12)	0.0016 (11)	−0.0023 (11)
C36	0.0139 (14)	0.0169 (13)	0.0248 (15)	0.0034 (11)	0.0050 (11)	0.0003 (11)
C62	0.0217 (15)	0.0281 (15)	0.0212 (15)	0.0027 (12)	0.0039 (12)	0.0021 (12)
C60	0.0259 (16)	0.0286 (16)	0.0219 (15)	0.0012 (13)	0.0028 (12)	0.0012 (13)
C50	0.0241 (15)	0.0269 (15)	0.0218 (15)	0.0011 (12)	0.0006 (12)	−0.0047 (12)
C22	0.0143 (14)	0.0179 (14)	0.0272 (16)	−0.0007 (11)	0.0075 (12)	−0.0005 (11)
C40	0.0195 (14)	0.0254 (15)	0.0172 (14)	0.0003 (11)	0.0021 (11)	−0.0011 (11)
C53	0.0211 (15)	0.0280 (16)	0.0270 (17)	0.0036 (12)	0.0019 (12)	0.0001 (12)
C37	0.0158 (13)	0.0102 (12)	0.0295 (16)	0.0012 (10)	0.0054 (11)	0.0003 (11)
C42	0.0231 (15)	0.0290 (16)	0.0176 (14)	0.0013 (12)	0.0064 (11)	0.0005 (12)
C41	0.0191 (14)	0.0202 (14)	0.0206 (15)	0.0024 (11)	0.0030 (11)	0.0029 (11)
C21	0.0129 (13)	0.0157 (13)	0.0154 (13)	0.0024 (11)	0.0038 (10)	0.0006 (11)
C47	0.0239 (15)	0.0266 (15)	0.0198 (15)	0.0037 (12)	0.0020 (11)	−0.0034 (11)
C64	0.0304 (17)	0.0340 (17)	0.0237 (16)	0.0047 (14)	0.0033 (13)	−0.0076 (13)
C55	0.0187 (14)	0.0223 (14)	0.0229 (16)	0.0049 (11)	−0.0001 (12)	−0.0012 (11)
C52	0.0371 (19)	0.0279 (17)	0.0296 (18)	−0.0070 (14)	0.0044 (14)	0.0041 (13)
C63	0.0316 (17)	0.0341 (17)	0.0196 (16)	0.0034 (14)	0.0070 (13)	−0.0004 (13)
C44	0.0347 (18)	0.0234 (15)	0.0319 (18)	−0.0074 (13)	0.0052 (14)	−0.0007 (13)
C46	0.0219 (15)	0.0311 (16)	0.0218 (15)	0.0003 (13)	0.0034 (12)	0.0040 (12)
C43	0.0228 (15)	0.0260 (15)	0.0222 (15)	−0.0002 (12)	0.0035 (12)	−0.0037 (12)
C39	0.0256 (16)	0.0233 (15)	0.0205 (15)	0.0013 (12)	0.0053 (12)	0.0007 (12)
C32	0.0200 (14)	0.0115 (13)	0.0309 (17)	0.0023 (11)	0.0096 (12)	0.0016 (11)
C56	0.0258 (16)	0.0296 (16)	0.0194 (15)	0.0044 (13)	0.0023 (12)	−0.0015 (12)
C58	0.0369 (19)	0.0261 (16)	0.0303 (18)	−0.0032 (14)	−0.0025 (14)	−0.0027 (13)
C57	0.0275 (16)	0.0318 (17)	0.0254 (16)	0.0057 (13)	−0.0024 (13)	−0.0090 (13)
C66	0.038 (2)	0.0373 (19)	0.0331 (19)	−0.0040 (15)	0.0117 (15)	0.0101 (15)
C51	0.042 (2)	0.0318 (18)	0.0269 (18)	−0.0040 (15)	−0.0022 (15)	−0.0051 (14)
C45	0.0344 (18)	0.0333 (17)	0.0287 (18)	−0.0062 (14)	0.0100 (14)	0.0082 (13)
C65	0.0346 (19)	0.0261 (16)	0.0348 (19)	−0.0073 (13)	0.0054 (15)	−0.0003 (13)
C59	0.0352 (19)	0.0381 (19)	0.0317 (19)	−0.0014 (15)	0.0023 (14)	0.0119 (15)

### 4-(Dimethylamino)pyridin-1-ium 8-hydroxyquinoline-5-sulfonate; N,N-dimethylpyridin-4-amine Geometric parameters (Å, º)

**Table:**

S1—O5	1.455 (2)	C31—C32	1.406 (4)
S1—O4	1.459 (2)	C28—H28	0.9500
S1—O3	1.447 (2)	C33—C32	1.375 (4)
S1—C24	1.772 (3)	C61—H61	0.9500
S2—O7	1.452 (2)	C61—C62	1.409 (4)
S2—O9	1.448 (2)	C61—C60	1.373 (4)
S2—O8	1.440 (2)	C54—H54	0.9500
S2—C33	1.776 (3)	C54—C53	1.359 (4)
O10—C30	1.359 (3)	C54—C55	1.426 (4)
O10—H10	0.90 (4)	C36—H36	0.9500
O6—C21	1.350 (3)	C36—C37	1.410 (4)
O6—H6	0.84 (4)	C62—C63	1.414 (4)
N11—C29	1.367 (4)	C60—H60	0.9500
N11—C28	1.316 (4)	C50—H50	0.9500
N14—C43	1.358 (4)	C22—H22	0.9500
N14—C39	1.354 (4)	C22—C21	1.372 (4)
N14—H14	0.90 (2)	C40—H40	0.9500
N18—C53	1.353 (4)	C40—C41	1.425 (4)
N18—C57	1.359 (5)	C40—C39	1.351 (4)
N18—H18	0.90 (2)	C53—H53	0.9500
N12—C38	1.366 (3)	C37—H37	0.9500
N12—C37	1.315 (4)	C42—H42	0.9500
N17—C55	1.338 (4)	C42—C41	1.417 (4)
N17—C58	1.466 (4)	C42—C43	1.360 (4)
N17—C59	1.462 (4)	C47—H47	0.9500
N13—C41	1.334 (4)	C47—C46	1.375 (4)
N13—C44	1.467 (4)	C64—H64	0.9500
N13—C45	1.464 (4)	C64—C63	1.369 (5)
C25—C29	1.422 (4)	C55—C56	1.421 (4)
C25—C24	1.428 (4)	C52—H52A	0.9800
C25—C26	1.415 (4)	C52—H52B	0.9800
N16—C48	1.356 (4)	C52—H52C	0.9800
N16—C52	1.458 (4)	C63—H63	0.9500
N16—C51	1.461 (4)	C44—H44A	0.9800
N15—C50	1.342 (4)	C44—H44B	0.9800
N15—C46	1.350 (4)	C44—H44C	0.9800
C30—C31	1.372 (4)	C46—H46	0.9500
C30—C38	1.423 (4)	C43—H43	0.9500
N20—C62	1.362 (4)	C39—H39	0.9500
N20—C66	1.453 (4)	C32—H32	0.9500
N20—C65	1.452 (4)	C56—H56	0.9500
C29—C21	1.424 (4)	C56—C57	1.348 (5)
N19—C60	1.342 (4)	C58—H58A	0.9800
N19—C64	1.350 (4)	C58—H58B	0.9800
C35—H35	0.9500	C58—H58C	0.9800
C35—C34	1.419 (4)	C57—H57	0.9500
C35—C36	1.365 (4)	C66—H66A	0.9800
C48—C49	1.407 (4)	C66—H66B	0.9800
C48—C47	1.413 (4)	C66—H66C	0.9800
C23—H23	0.9500	C51—H51A	0.9800
C23—C24	1.367 (4)	C51—H51B	0.9800
C23—C22	1.408 (4)	C51—H51C	0.9800
C27—H27	0.9500	C45—H45A	0.9800
C27—C26	1.365 (4)	C45—H45B	0.9800
C27—C28	1.407 (4)	C45—H45C	0.9800
C26—H26	0.9500	C65—H65A	0.9800
C34—C38	1.419 (4)	C65—H65B	0.9800
C34—C33	1.418 (4)	C65—H65C	0.9800
C49—H49	0.9500	C59—H59A	0.9800
C49—C50	1.377 (4)	C59—H59B	0.9800
C31—H31	0.9500	C59—H59C	0.9800
			
N14···H14	0.84		
			
O5—S1—O4	111.94 (13)	C23—C22—H22	120.1
O5—S1—C24	107.15 (12)	C21—C22—C23	119.9 (3)
O4—S1—C24	104.71 (13)	C21—C22—H22	120.1
O3—S1—O5	112.26 (14)	C41—C40—H40	119.3
O3—S1—O4	113.97 (13)	C39—C40—H40	119.3
O3—S1—C24	106.09 (13)	C39—C40—C41	121.3 (3)
O7—S2—C33	106.42 (13)	N18—C53—C54	122.3 (3)
O9—S2—O7	111.50 (14)	N18—C53—H53	118.9
O9—S2—C33	106.67 (13)	C54—C53—H53	118.9
O8—S2—O7	112.15 (17)	N12—C37—C36	123.7 (2)
O8—S2—O9	114.36 (16)	N12—C37—H37	118.2
O8—S2—C33	105.04 (14)	C36—C37—H37	118.2
C30—O10—H10	114 (3)	C41—C42—H42	119.6
C21—O6—H6	107 (3)	C43—C42—H42	119.6
C28—N11—C29	117.2 (2)	C43—C42—C41	120.7 (3)
C43—N14—H14	115 (2)	N13—C41—C40	121.9 (3)
C39—N14—C43	119.4 (3)	N13—C41—C42	122.6 (3)
C39—N14—H14	125 (2)	C42—C41—C40	115.4 (3)
C53—N18—C57	119.1 (3)	O6—C21—C29	120.6 (2)
C53—N18—H18	121 (3)	O6—C21—C22	119.3 (3)
C57—N18—H18	120 (3)	C22—C21—C29	120.2 (3)
C37—N12—C38	117.4 (2)	C48—C47—H47	120.0
C55—N17—C58	121.4 (3)	C46—C47—C48	120.0 (3)
C55—N17—C59	120.8 (3)	C46—C47—H47	120.0
C59—N17—C58	117.6 (3)	N19—C64—H64	117.6
C41—N13—C44	120.9 (3)	N19—C64—C63	124.9 (3)
C41—N13—C45	121.3 (3)	C63—C64—H64	117.6
C45—N13—C44	117.7 (3)	N17—C55—C54	121.7 (3)
C29—C25—C24	118.4 (2)	N17—C55—C56	122.7 (3)
C26—C25—C29	116.6 (2)	C56—C55—C54	115.6 (3)
C26—C25—C24	125.0 (2)	N16—C52—H52A	109.5
C48—N16—C52	120.9 (3)	N16—C52—H52B	109.5
C48—N16—C51	121.8 (3)	N16—C52—H52C	109.5
C52—N16—C51	117.1 (3)	H52A—C52—H52B	109.5
C50—N15—C46	115.2 (3)	H52A—C52—H52C	109.5
O10—C30—C31	119.3 (2)	H52B—C52—H52C	109.5
O10—C30—C38	120.1 (2)	C62—C63—H63	120.1
C31—C30—C38	120.6 (2)	C64—C63—C62	119.8 (3)
C62—N20—C66	120.6 (3)	C64—C63—H63	120.1
C62—N20—C65	120.8 (3)	N13—C44—H44A	109.5
C65—N20—C66	118.5 (3)	N13—C44—H44B	109.5
N11—C29—C25	123.4 (3)	N13—C44—H44C	109.5
N11—C29—C21	116.8 (2)	H44A—C44—H44B	109.5
C25—C29—C21	119.8 (2)	H44A—C44—H44C	109.5
C60—N19—C64	115.2 (3)	H44B—C44—H44C	109.5
C34—C35—H35	120.5	N15—C46—C47	124.5 (3)
C36—C35—H35	120.5	N15—C46—H46	117.7
C36—C35—C34	118.9 (3)	C47—C46—H46	117.7
N16—C48—C49	122.2 (3)	N14—C43—C42	121.7 (3)
N16—C48—C47	122.4 (3)	N14—C43—H43	119.1
C49—C48—C47	115.4 (3)	C42—C43—H43	119.1
C24—C23—H23	119.2	N14—C39—H39	119.3
C24—C23—C22	121.7 (2)	C40—C39—N14	121.4 (3)
C22—C23—H23	119.2	C40—C39—H39	119.3
C25—C24—S1	120.6 (2)	C31—C32—H32	119.3
C23—C24—S1	119.4 (2)	C33—C32—C31	121.4 (2)
C23—C24—C25	120.0 (2)	C33—C32—H32	119.3
C26—C27—H27	120.4	C55—C56—H56	119.4
C26—C27—C28	119.2 (3)	C57—C56—C55	121.2 (3)
C28—C27—H27	120.4	C57—C56—H56	119.4
C25—C26—H26	120.2	N17—C58—H58A	109.5
C27—C26—C25	119.6 (3)	N17—C58—H58B	109.5
C27—C26—H26	120.2	N17—C58—H58C	109.5
C35—C34—C38	117.1 (2)	H58A—C58—H58B	109.5
C33—C34—C35	124.2 (2)	H58A—C58—H58C	109.5
C33—C34—C38	118.7 (2)	H58B—C58—H58C	109.5
C48—C49—H49	120.0	N18—C57—H57	119.2
C50—C49—C48	119.9 (3)	C56—C57—N18	121.7 (3)
C50—C49—H49	120.0	C56—C57—H57	119.2
C30—C31—H31	120.2	N20—C66—H66A	109.5
C30—C31—C32	119.7 (3)	N20—C66—H66B	109.5
C32—C31—H31	120.2	N20—C66—H66C	109.5
N12—C38—C30	117.2 (2)	H66A—C66—H66B	109.5
N12—C38—C34	123.3 (3)	H66A—C66—H66C	109.5
C34—C38—C30	119.5 (2)	H66B—C66—H66C	109.5
N11—C28—C27	124.0 (3)	N16—C51—H51A	109.5
N11—C28—H28	118.0	N16—C51—H51B	109.5
C27—C28—H28	118.0	N16—C51—H51C	109.5
C34—C33—S2	120.5 (2)	H51A—C51—H51B	109.5
C32—C33—S2	119.3 (2)	H51A—C51—H51C	109.5
C32—C33—C34	120.2 (3)	H51B—C51—H51C	109.5
C62—C61—H61	119.9	N13—C45—H45A	109.5
C60—C61—H61	119.9	N13—C45—H45B	109.5
C60—C61—C62	120.1 (3)	N13—C45—H45C	109.5
C53—C54—H54	119.9	H45A—C45—H45B	109.5
C53—C54—C55	120.2 (3)	H45A—C45—H45C	109.5
C55—C54—H54	119.9	H45B—C45—H45C	109.5
C35—C36—H36	120.2	N20—C65—H65A	109.5
C35—C36—C37	119.6 (3)	N20—C65—H65B	109.5
C37—C36—H36	120.2	N20—C65—H65C	109.5
N20—C62—C61	122.2 (3)	H65A—C65—H65B	109.5
N20—C62—C63	122.4 (3)	H65A—C65—H65C	109.5
C61—C62—C63	115.4 (3)	H65B—C65—H65C	109.5
N19—C60—C61	124.6 (3)	N17—C59—H59A	109.5
N19—C60—H60	117.7	N17—C59—H59B	109.5
C61—C60—H60	117.7	N17—C59—H59C	109.5
N15—C50—C49	124.9 (3)	H59A—C59—H59B	109.5
N15—C50—H50	117.6	H59A—C59—H59C	109.5
C49—C50—H50	117.6	H59B—C59—H59C	109.5
			
S2—C33—C32—C31	−178.5 (2)	C38—C30—C31—C32	−1.1 (4)
O5—S1—C24—C25	50.1 (3)	C38—C34—C33—S2	178.2 (2)
O5—S1—C24—C23	−131.4 (2)	C38—C34—C33—C32	−0.5 (4)
O10—C30—C31—C32	178.2 (3)	C28—N11—C29—C25	0.5 (4)
O10—C30—C38—N12	1.2 (4)	C28—N11—C29—C21	−179.2 (3)
O10—C30—C38—C34	−178.4 (2)	C28—C27—C26—C25	0.5 (4)
O4—S1—C24—C25	169.1 (2)	C33—C34—C38—N12	−179.6 (3)
O4—S1—C24—C23	−12.4 (3)	C33—C34—C38—C30	0.0 (4)
O7—S2—C33—C34	−57.5 (3)	C61—C62—C63—C64	−0.7 (5)
O7—S2—C33—C32	121.2 (3)	C54—C55—C56—C57	1.3 (4)
N11—C29—C21—O6	0.1 (4)	C36—C35—C34—C38	0.5 (4)
N11—C29—C21—C22	−179.9 (3)	C36—C35—C34—C33	179.4 (3)
O3—S1—C24—C25	−70.0 (3)	C62—C61—C60—N19	−1.2 (5)
O3—S1—C24—C23	108.5 (2)	C60—N19—C64—C63	−0.5 (5)
O9—S2—C33—C34	61.6 (3)	C60—C61—C62—N20	−178.7 (3)
O9—S2—C33—C32	−119.6 (3)	C60—C61—C62—C63	1.1 (5)
N17—C55—C56—C57	−179.3 (3)	C50—N15—C46—C47	−0.9 (5)
C25—C29—C21—O6	−179.6 (3)	C22—C23—C24—S1	−177.6 (2)
C25—C29—C21—C22	0.4 (4)	C22—C23—C24—C25	0.9 (4)
N16—C48—C49—C50	178.0 (3)	C53—N18—C57—C56	−0.4 (5)
N16—C48—C47—C46	−178.6 (3)	C53—C54—C55—N17	−179.8 (3)
C30—C31—C32—C33	0.6 (5)	C53—C54—C55—C56	−0.4 (4)
N20—C62—C63—C64	179.1 (3)	C37—N12—C38—C30	−179.2 (3)
C29—N11—C28—C27	0.4 (4)	C37—N12—C38—C34	0.5 (4)
C29—C25—C24—S1	178.2 (2)	C41—C40—C39—N14	0.4 (5)
C29—C25—C24—C23	−0.3 (4)	C41—C42—C43—N14	−0.4 (5)
C29—C25—C26—C27	0.4 (4)	C47—C48—C49—C50	−1.9 (4)
N19—C64—C63—C62	0.4 (5)	C64—N19—C60—C61	0.9 (5)
C35—C34—C38—N12	−0.7 (4)	C55—C54—C53—N18	−0.8 (5)
C35—C34—C38—C30	179.0 (3)	C55—C56—C57—N18	−0.9 (5)
C35—C34—C33—S2	−0.7 (4)	C52—N16—C48—C49	−4.7 (5)
C35—C34—C33—C32	−179.4 (3)	C52—N16—C48—C47	175.2 (3)
C35—C36—C37—N12	−0.1 (5)	C44—N13—C41—C40	−0.5 (5)
O8—S2—C33—C34	−176.6 (2)	C44—N13—C41—C42	179.4 (3)
O8—S2—C33—C32	2.1 (3)	C46—N15—C50—C49	0.3 (5)
C48—C49—C50—N15	1.2 (5)	C43—N14—C39—C40	0.8 (5)
C48—C47—C46—N15	0.1 (5)	C43—C42—C41—N13	−178.4 (3)
C23—C22—C21—O6	−179.8 (3)	C43—C42—C41—C40	1.6 (4)
C23—C22—C21—C29	0.2 (4)	C39—N14—C43—C42	−0.8 (5)
C24—C25—C29—N11	180.0 (3)	C39—C40—C41—N13	178.4 (3)
C24—C25—C29—C21	−0.3 (4)	C39—C40—C41—C42	−1.6 (4)
C24—C25—C26—C27	179.4 (3)	C58—N17—C55—C54	−2.7 (4)
C24—C23—C22—C21	−0.8 (4)	C58—N17—C55—C56	177.9 (3)
C26—C25—C29—N11	−0.9 (4)	C57—N18—C53—C54	1.2 (5)
C26—C25—C29—C21	178.7 (3)	C66—N20—C62—C61	174.1 (3)
C26—C25—C24—S1	−0.8 (4)	C66—N20—C62—C63	−5.7 (5)
C26—C25—C24—C23	−179.3 (3)	C51—N16—C48—C49	179.8 (3)
C26—C27—C28—N11	−0.9 (5)	C51—N16—C48—C47	−0.3 (5)
C34—C35—C36—C37	−0.1 (4)	C45—N13—C41—C40	−176.3 (3)
C34—C33—C32—C31	0.2 (5)	C45—N13—C41—C42	3.7 (5)
C49—C48—C47—C46	1.3 (4)	C65—N20—C62—C61	−3.0 (5)
C31—C30—C38—N12	−179.6 (3)	C65—N20—C62—C63	177.2 (3)
C31—C30—C38—C34	0.8 (4)	C59—N17—C55—C54	−177.4 (3)
C38—N12—C37—C36	−0.1 (4)	C59—N17—C55—C56	3.2 (5)

### 4-(Dimethylamino)pyridin-1-ium 8-hydroxyquinoline-5-sulfonate; N,N-dimethylpyridin-4-amine Hydrogen-bond geometry (Å, º)

*Cg*1, *Cg*2 are the centroids of the N11/C25–C29 and N12/C34–C38 rings, respectively

**Table:**

*D*—H···*A*	*D*—H	H···*A*	*D*···*A*	*D*—H···*A*
O6—H6···O8^i^	0.84 (5)	2.00 (5)	2.679 (3)	137 (4)
O6—H6···N11	0.84 (5)	2.24 (5)	2.728 (3)	117 (4)
O10—H10···O4	0.89 (5)	1.90 (5)	2.674 (3)	144 (4)
O10—H10···N12	0.89 (5)	2.31 (5)	2.730 (3)	109 (4)
N14—H14···N15	0.90 (3)	1.91 (3)	2.814 (4)	174 (3)
N18—H18···N19	0.90 (3)	1.92 (4)	2.816 (4)	177 (7)
C27—H27···O6^ii^	0.95	2.58	3.219 (4)	125
C27—H27···O8^iii^	0.95	2.27	3.194 (4)	164
C36—H36···O4^iv^	0.95	2.33	3.244 (4)	160
C36—H36···O10^iv^	0.95	2.58	3.216 (4)	125
C39—H39···O3^v^	0.95	2.32	3.200 (4)	154
C43—H43···O5	0.95	2.22	3.160 (4)	169
C46—H46···O5	0.95	2.46	3.373 (4)	161
C50—H50···O3^v^	0.95	2.43	3.292 (4)	151
C53—H53···O9^vi^	0.95	2.22	3.146 (4)	164
C54—H54···O10^vii^	0.95	2.55	3.450 (4)	158
C57—H57···O7	0.95	2.22	3.143 (4)	165
C60—H60···O7	0.95	2.40	3.325 (4)	163
C64—H64···O9^vi^	0.95	2.35	3.267 (4)	162
C40—H40···*Cg*1^v^	0.95	2.63	3.498 (3)	153
C49—H49···*Cg*2^viii^	0.95	2.87	3.754 (3)	156
C61—H61···*Cg*2	0.95	2.70	3.571 (3)	152

Symmetry codes: (i) *x*, *y*−1, *z*; (ii) *x*−1, *y*, *z*; (iii) *x*−1, *y*−1, *z*; (iv) *x*+1, *y*, *z*; (v) *x*, −*y*+1, *z*+1/2; (vi) *x*, −*y*+2, *z*+1/2; (vii) *x*+1, −*y*+2, *z*+1/2; (viii) *x*−1, −*y*+1, *z*+1/2.
